# Research on the Integration of Sensing and Communication Based on Fractional-Order Fourier Transform

**DOI:** 10.3390/s25102956

**Published:** 2025-05-08

**Authors:** Mingyan Qi, Yuelong Su, Zhaoyi Wang, Kun Lu

**Affiliations:** 1 Nanjing Research Institute of Electronics Technology, Nanjing 210039, Chinastan_wzy@163.com (Z.W.); 2 The College of Urban & Environmental Sciences, Central China Normal University, Wuhan 430079, China; mersuyl@mails.ccnu.edu.cn; 3 Key Laboratory for Geographical Process Analysis & Simulation in Hubei Province, Central China Normal University, Wuhan 430079, China

**Keywords:** integration of sensing and communication, LFM, fractional-order fourier transform, anti-frequency sweeping interference

## Abstract

This study investigated the integration of detection and communication techniques. First, the fractional-order Fourier transform (FRFT) is introduced, and the golden section method, parabolic interpolation, and Brent method are applied to search for the optimal fractional-order domain to accurately estimate the parameters of the linear frequency modulation (LFM) signal. Second, the three search algorithms and the performance of the integrated sensing and communication waveform are simulated. The Brent method improves the parameter searching efficiency by approximately 30% compared with the golden section method; the bit error ratio (BER) of the integrated LFM signal can reach 10^−4^ with a signal-to-noise ratio (SNR) of 3 dB. The results show that the integrated waveform can realize the detection function with guaranteed communication performance. An anti-frequency sweeping interference method based on the fractional domain matching order was also carried out to optimize the detection performance of the integrated waveform. Through the analysis of the difference-frequency signal under frequency sweeping interference, two methods, direct filtering, and pairwise cancellation filtering, are used to suppress the interference signal and detect the target distance. The simulation evaluated the detection performance of the two methods under different signal-to-interference ratios (SIR) and filter widths. The simulation results show that the pairwise cancellation filtering suppresses the frequency sweeping interference by 4–6 dB more than the direct filtering with an SIR ≤ −15 dB. Both filtering methods can correctly extract the target position information under frequency sweeping interference with a low signal-to-interference ratio (SIR). In conclusion, this study provides an effective solution for parameter estimation optimization and frequency-sweeping interference suppression for FRFT-based sensing communication systems.

## 1. Introduction

In today’s digital era, with the wide application of wireless communication and radar detection technologies, spectrum resources are becoming tighter, and the requirements for system integration and performance are increasing. As a key method to solve these problems, detection and communication integration technology has become a popular research topic in both academia and industry. This technology aims to build a system that can simultaneously perform detection and communication functions, breaking the traditional architecture in which detection and communication are independent of each other, realizing the efficient utilization of resources and the improvement of system performance, and shows great potential for application in many fields, such as military and civil engineering. The sensing and communication integration strategy has application prospects in the fields of unmanned aerial vehicles [1], industrial robotics [2], automated systems, smart cities, and the industrial Internet of Things [3]. In some vehicle-mounted radar communication integrated systems [4,5], the sensing and communication integrated signal can detect the distance and speed of the surrounding targets, and at the same time, it can also send the vehicle’s driving status, position, and other information in the form of communication data, which can provide key support for the intelligent transportation system.

In terms of signal design, many types of signals are used in integrated sensing communication systems. Linear frequency modulation (LFM) signals were one of the earlier applications that had an important position. LFM signals have a large time-width bandwidth product by changing the frequency linearly over the pulse duration, which can improve the radar range resolution without increasing the peak power. Zhu et al. proposed a long-range target detection method based on broadband frequency modulated continuous wave (FMCW) radar [6]. The research results of LFM-CW radar have been validated in the universal software radio peripheral (USRP N210) to achieve 70 km range detection [7]. The LFM signal exhibited a high peak power characteristic under the optimal fractional-order Fourier transform (FRFT) [8]. On the basis of the discrete fractional-order Fourier transform—orthogonal frequency division multiplexing (DFRFT-OFDM) waveform, a DFRFT-OFDM waveform-based distance estimation algorithm is based on pulse compression and a velocity estimation algorithm is based on coherent accumulation [9]. Using the matching property of LFM signals in the fractional-order domain, signal detection [10,11] and radar sonar imaging [12] can be realized. In sensing communication integration scenarios, LFM signals can be utilized for target detection with good autocorrelation properties while loading communication information by modulating the signals appropriately. The time-domain matching compression (TDMC) technique for LFM signals has the unique advantage of effectively reducing the signal power spectral density in the transmission process while realizing signal detection through the energy-focusing effect, which is in line with the intrinsic safety and explosion-proof standards of mines. On the basis of this feature, the LFM-TDMC binary quadrature keying (BOK) modulation scheme [13] has been developed as an ideal choice for coal mine tunnel wireless communication systems owing to its low power spectral density and reliable signal detection capability. Orthogonal frequency division multiplexing (OFDM) signals have attracted considerable attention in recent years in the fields of sensing and communication integration. OFDM technology assigns high-speed data streams to multiple low-speed subcarriers for transmission through serial-to-parallel conversion, and the subcarriers are orthogonal to each other, which effectively improves spectrum utilization. In terms of communication, its multicarrier modulation characteristics make it flexible for loading different communication data on different subcarriers. In terms of the sensing function, OFDM signals can obtain information such as the distance, speed, and angle of the target by transmitting subcarriers with different frequencies [14,15,16]. Considering the research on the integration of 5G communication and radar sensing as an example, OFDM signals, with their advantages of strong resistance to multipath fading and high spectrum utilization, can not only satisfy the demand for 5G communication for high-speed rate and large-capacity data transmission but also realize accurate detection of surrounding targets through signal processing. Constant envelope technology was introduced on the basis of the orthogonal frequency division multiplexed linear frequency modulation (OFDM-LFM) radar system to generate pulse waveforms on the basis of time-frequency modulation, which meets the requirements of star-ground communication and radar sensing [17]. The MIMO SAR system based on OFDM-LFM achieves a higher SNR and better SAR image distance resolution [15]. The OFDM-LFM signal parameter estimation algorithm [18] provides a feasible technical route for the realization and performance enhancement of sensing–communication integration.

With the continuous development of technology, the integrated technology of sensing and communication has made significant progress in terms of algorithm optimization and system integration. At the algorithmic level, joint signal-processing algorithms continue to emerge to better integrate sensing and communication functions. For example, joint estimation algorithms based on compressed sensing can simultaneously and accurately estimate the signal arrival direction and frequency information in low SNR environments to improve the overall performance of the system [19]. In unmanned aerial vehicle (UAV) radar-communication integrated networks, resource allocation techniques are essential to improve the detection and communication performance of the system. Zhang et al. proposed a strategy for joint target and user assignment, power and bandwidth allocation, and subchannel allocation so as to adaptively adjust the resource allocation and improve the efficiency of system resource utilization [20,21]. In terms of system integration, an increasing number of studies have focused on the integrated design of sensing and communication hardware to reduce the system size, weight, and power consumption and improve the applicability and deployability of the system. For example, some new RF front-end designs can realize the transmission and reception of multiple signals on the same hardware platform and flexibly switch between sensing and communication modes.

However, integrated sensing and communication technologies still face several challenges. Different application scenarios have different requirements for sensing and communication performance. Therefore, balancing the performance of both in the same system and realizing the optimal allocation of resources is a problem that needs to be solved. However, as the electromagnetic environment becomes increasingly complex, improving the system’s anti-jamming ability and ensuring the reliability of sensing and communication functions are also key directions of research in this field. Zhu et al. and Yin et al. based on a modified Eckart filter for scanning azimuth, compared with the traditional beam forming method, it can improve the output SNR and angular resolution, which effectively enhances the detection capability of underwater weak targets [22,23]. Xu et al. improve the detection performance of extended targets through the joint design of transmit waveform and receive filter [24]. Zhu et al. proposed the oversampled generalized Prouhet-Thue-Morse (OGPTM) method to design the complementary set of transmit signals and by pointwise multiplication processor (PMuP) integrating the delay-Doppler maps of two oversampled complementary sets [25]. This method provides better sidelobe-valve suppression performance in high-speed target detection.

**Figure 1:**
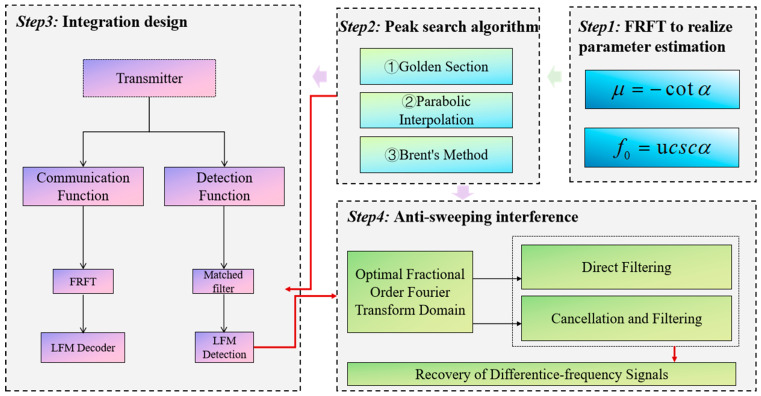
Research framework of this paper.

The research framework of this study is illustrated in Figure 1. This section describes some of the signals and related techniques applied in the design of detection and communication integration and provides a brief overview of anti-jamming techniques for integrated detection and communication systems. This section emphasizes the need for detection and communication integration in the context of tight spectrum resources and increased requirements for the integration of system structures and functions. It also illustrates the value of anti-jamming techniques for improving the performance of integrated systems. Subsequently, Section 2.1 introduces the FRFT, and Section 2.2 utilizes the golden section, parabolic interpolation, and Brent method for the optimal fractional order domain search. These algorithms enable communication functions based on LFM signals to quickly estimate the chirp rate of received LFM signals and then demodulate the recovered data. Section 2.3 presents the study of the anti-jamming method, which is an algorithm that can improve the detection performance of LFM signals under frequency sweeping interference. By analyzing the difference frequency signal under the frequency sweeping interference, the aforementioned FRFT, and search algorithm are used to find the optimal transform order to achieve the suppression of the interference signal and the detection of the target distance. In Section 3.1, the performance of the three search algorithms is simulated and compared. In Section 3.2, an integrated frame structure for LFM detection and communication is designed. In Section 3.3, the performance of the integrated waveform is simulated, which mainly focuses on the BER in communication and the fuzzy function in detection. Section 3.4 simulates the anti-jamming method to improve the detection performance and uses both direct filtering and pair-cancellation filtering to realize the target distance detection of LFM fuzes under frequency sweeping interference, and the simulation results show that the anti-jamming performance of pair-cancellation filtering is more superior, and the reliability of the detection results is also higher. The simulation results are discussed in Section 4, which proves that the Brent method improves the search efficiency, the integrated system realized based on the search algorithm can realize the communication and detection functions at the same time, and the anti-jamming algorithm proposed in this paper improves the detection performance significantly. This section also points out the deficiencies and directions for improvement in this research. Section 5 summarizes the research results of this paper and gives an outlook on the development of the integration of detection and communication.

Overall, the development prospects of sensing and communication integration technology are broad; research and applications based on LFM signals provide important support for its development, while the exploration of other types of signals also energizes the diversified development of this technology. With continuous innovation and breakthroughs in technology, integrated sensing, and communication technology is expected to be widely used in more fields and to promote the change and development of related industries.

## 2. Related Technologies

### 2.1. Realization of LFM Parameter Estimation Based on FRFT

The FRFT is a generalized form of the Fourier transform, which rotates the projection of a signal in the time-frequency plane. The basic mathematical formula is as follows:

(1) $$F^{p}(u)=\int_{-\infty }^{\infty }f(t)K_{p}(t,u)dt$$

where $F^{p}(u)$ is the result of the p order FRFT of the signal f(t), and $K_{p}(t,u)$ is the kernel function of the FRFT and is expressed as follows:

(2) $$K_{p}(t,u)=\left\{\begin{array}{ll} \sqrt{\frac{1-j\cot\alpha }{2\pi }}\exp\left(j\frac{t^{2}+u^{2}}{2}\cot\alpha -jtu\csc\alpha \right), & \alpha \neq n\pi \\ \delta (t-u), & \alpha =2n\pi \\ \delta (t+u), & \begin{array}{l} \alpha (2n+1)\pi \end{array} \end{array}\right.$$

Here, α=pπ/2, and n is an integer. When α≠nπ, the kernel function $\sqrt{\frac{1-j\cot\alpha }{2\pi }}$ is a normalization coefficient in the kernel function to ensure the energy conservation properties of the transform. Then, $\exp\left(j\frac{t^{2}+u^{2}}{2}\cot\alpha -jtu\csc\alpha \right)$ embodies the rotation and phase change in the signal in the time-frequency plane. When α=2nπ, the kernel function becomes δ(t−u),; at this time, the FRFT degenerates into a constant transform $F^{p}(u)=f(u)$, and the signal remains unchanged in the time domain. When α=(2n+1)π, the kernel function is δ(t+u), which is a special form of the transform and is related to the characteristics of the signal, such as parity.

The expression of the LFM signal is usually:

(3) $$f(t)=A\exp\left[j(2\pi f_{0}t+\frac{1}{2}\mu t^{2})\right]$$

where A denotes the amplitude, $f_{0}$ denotes the initial frequency, and μ denotes the chirp rate.

Substituting the LFM signal f(t) into the FRFT formula, the exponential term containing the integral variable t is:

(4) $$\begin{array}{l} \varphi (t)=j2\pi \left(\frac{\mu }{2}t^{2}+f_{0}t\right)+j\pi t^{2}\cot\alpha -j2\pi tu\csc\alpha \\ =j\pi t^{2}\left(\mu +\cot\alpha \right)+j2\pi t\left(f_{0}-u\csc\alpha \right) \end{array}$$

Let the coefficient of the second-order term of t be 0, the peak of the amplitude appears, and the FRFT is used to estimate the LFM chirp rate $\hat{\mu }$. By traversing different fractional orders, the optimal fractional order was determined to maximize the amplitude. Because $\hat{\mu }+\cot\alpha =0$, the estimated value of the chirp rate is

(5) $$\hat{\mu }=-\cot\alpha$$

Because the energy aggregation point u satisfies ${\hat{f}}_{0}-u\csc\alpha =0$ at the optimal fractional order, the estimate of the center frequency ${\hat{f}}_{0}$ is as follows:

(6) $${\hat{f}}_{0}=u\csc\alpha$$

The LFM signal has an energy aggregation property in a specific fractional-order Fourier domain [26,27,28], and when the fractional order α=arctan(−1/μ) is satisfied, the signal energy is concentrated at one point. The optimal fractional order is determined by searching the maximum point of the FRFT results under different fractional orders, and the chirp rate μ and center frequency $f_{0}$ of the LFM signal can be estimated. This method utilizes the time-frequency characteristics of the LFM signal and the rotational nature of the FRFT, which can effectively realize parameter estimation in an environment with a low SNR.

**Figure 2:**
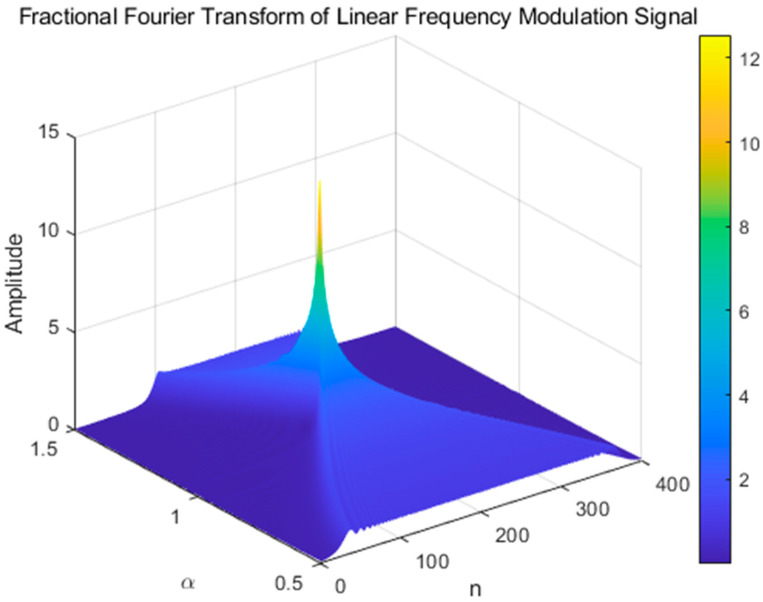
Fractional Fourier transform of the LFM signal.

The peaks of the FRFT spectrum at different orders were analyzed and plotted in two dimensions. Figure 2 shows the variation in the peaks with the fractional order, which provides a more accurate analysis basis for determining the optimal order on the basis of the peak search and the estimation of the LFM signal parameters.

**Figure 3:**
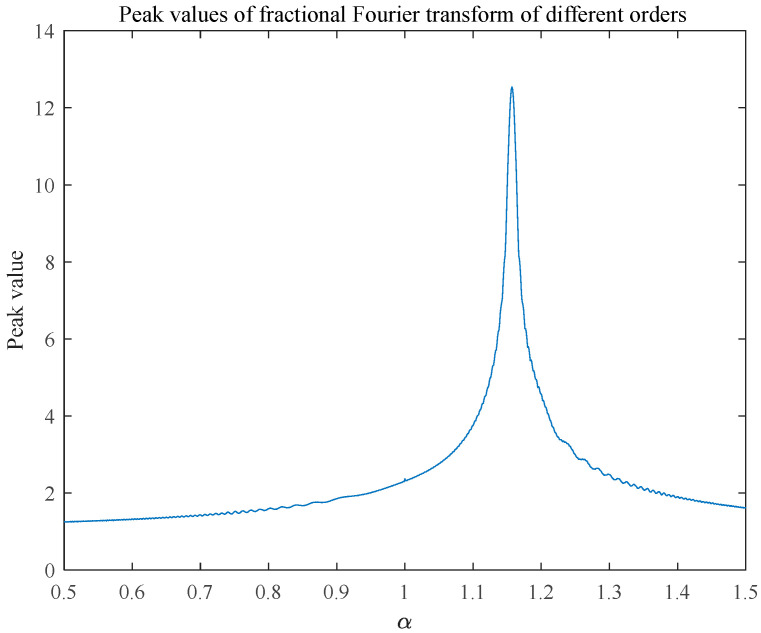
FRFT peak of different orders.

Figure 3 illustrates the peaks in the FRFT domain at different transform orders. The energy aggregation property in the FRFT domain effectively estimates parameters such as the chirp rate and center frequency of the LFM signal. This method provides key theoretical support for the accurate analysis and processing of LFM signals via integrated waveform technology, which helps improve the sensing and communication performance of radar systems.

### 2.2. Optimized Fractional Order Domain Search Algorithm

In the spectral analysis of LFM signals via the FRFT, an accurate search of the peaks in the fractional frequency domain is crucial for estimating the parameters of the LFM signals (e.g., chirp rate and center frequency). In this chapter, the principles of two peak search algorithms, the golden section method, and the parabolic interpolation method, are introduced in detail. The Brent search algorithm is proposed, and its performance advantages and disadvantages are compared to provide a theoretical basis for choosing the appropriate algorithms for parameter estimation of LFM signals.

#### 2.2.1. Golden Section Method

The golden section method [29,30] is an iterative algorithm based on interval elimination that is used to find the extreme value points of a function in a given interval. The core idea is to gradually approximate the true extreme value location by continuously shrinking the interval that contains the extreme value point. In fractional frequency domain peak search, suppose that we want to find the peak of the function f(x) within the interval $\left[a,b\right]$; at each iteration, two points $x_{1}$ and $x_{2}$ are selected within the interval, dividing the interval into three segments.

The golden section points were selected on the basis of the golden section ratio $\varphi =\sqrt{5}-1/2\approx 0.618$. Let $a_{1}=a+(1-\varphi )(b-a)$, $b_{1}=a+\varphi (b-a)$. The values of $f\left(a_{1}\right)$ and $f\left(b_{1}\right)$ are calculated. If $f\left(a_{1}\right)>f\left(b_{1}\right)$, then the new search interval is $\left[a,b_{1}\right]$; if $f\left(a_{1}\right)<f\left(b_{1}\right)$, the new search interval is $\left[a_{1},b\right]$. This process is repeated until the length of the interval is less than the preset accuracy threshold ϵ, at which point the midpoint within the interval can be approximated as the peak point.

**Figure 4:**
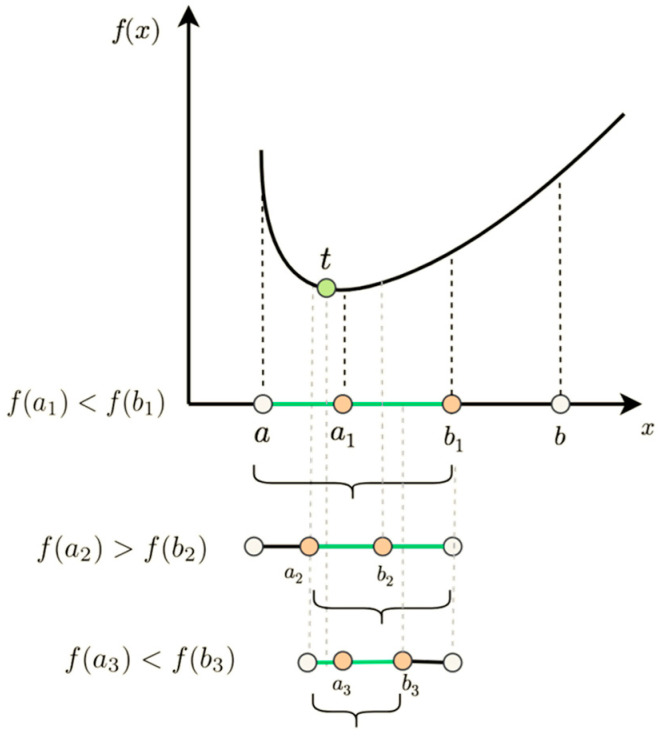
Principle diagram of the golden section method.

The principle of the golden sectioning method is shown in Figure 4. In the figure, the initial interval is $\left[a,b\right]$ through the golden section ratio to determine the two inner points $a_{1}$ and $b_{1}$. According to the relationship between the sizes of the function values $f\left(a_{1}\right)$ and $f\left(b_{1}\right)$, the size of the interval constantly shrinks and gradually approaches the peak point.

The golden section method is simple in principle, easy to implement, does not need to calculate the derivative of the function, and is applicable to a variety of complex functions. Moreover, it has a fast convergence speed, and each iteration can shorten the interval length by approximately 0.382, which can approach the peak point in a small number of iterations.

However, its convergence speed is relatively slow, especially near the peak point, and the convergence speed gradually decreases. When there are multiple local extremes in the function, the golden section method can find only one extreme point in the initial interval and cannot guarantee the identification of the global maximum.

#### 2.2.2. Parabolic Interpolation

Parabolic interpolation [31,32] is an iterative algorithm based on the local quadratic approximation of a function. It utilizes the values of the function at three points to construct a quadratic parabola to approximate the original function, and the peak points are then estimated by solving the vertices of the parabola. In the fractional frequency domain peak search, three points, $x_{1}$, $x_{2}$ and $x_{3}$ (usually $x_{1}<x_{2}<x_{3}$), are first selected, and the corresponding function values are $f(x_{1})$, $f(x_{2})$, and $f(x_{3})$.

A quadratic parabola $y=ax^{2}+bx+c$ is constructed from these three points and by solving the system of equations

(7) $$\left\{\begin{array}{l} f(x_{1})=ax_{1}^{2}+bx_{1}+c\\ f(x_{2})=ax_{2}^{2}+bx_{2}+c\\ f(x_{3})=ax_{3}^{2}+bx_{3}+c \end{array}\right.$$

The coefficients of the parabolas a, b, c can be obtained. The horizontal coordinates of the vertices $x_{new}=-b/2a$ of the parabola are new peak estimation points. Replace one of the points in $x_{1}$, $x_{2}$, $x_{3}$ with a replacement $x_{new}$ (usually replacing the point farthest from $x_{new}$), and the process is repeated until the convergence condition is satisfied.

Let the constructed quadratic parabola be $y=ax^{2}+bx+c$, which is obtained from the values of the functions at three points:

(8) $$\left\{\begin{array}{l} a=\frac{\left(x_{3}-x_{2})(f(x_{1})-f(x_{2}))-(x_{2}-x_{1})(f(x_{2})-f(x_{3})\right)}{\left(x_{1}-x_{2})(x_{2}-x_{3})(x_{1}-x_{3}\right)}\\ \begin{array}{l} b=\frac{\left(x_{3}^{2}-x_{2}^{2})(f(x_{1})-f(x_{2}))-(x_{2}^{2}-x_{1}^{2})(f(x_{2})-f(x_{3})\right)}{\left(x_{1}-x_{2})(x_{2}-x_{3})(x_{1}-x_{3}\right)} \end{array}\\ \begin{array}{l} c=f(x_{1})-ax_{1}^{2}-bx_{1} \end{array} \end{array}\right.$$

New peak estimation point $x_{new}=-b/2a$.

Derived from the Lagrange interpolation method, computing the min/max points of the parabolas is straightforward.

(9) $$x_{\mathrm{new}}=x_{2}-\frac{{\left(x_{2}-x_{1}\right)}^{2}[f(x_{2})-f(x_{3})]-{\left(x_{2}-x_{3}\right)}^{2}[f(x_{2})-f(x_{1})]}{2\left[\left(x_{2}-x_{1})(f(x_{2})-f(x_{3}))-(x_{2}-x_{3})(f(x_{2})-f(x_{1})\right)\right]}$$

**Figure 5:**
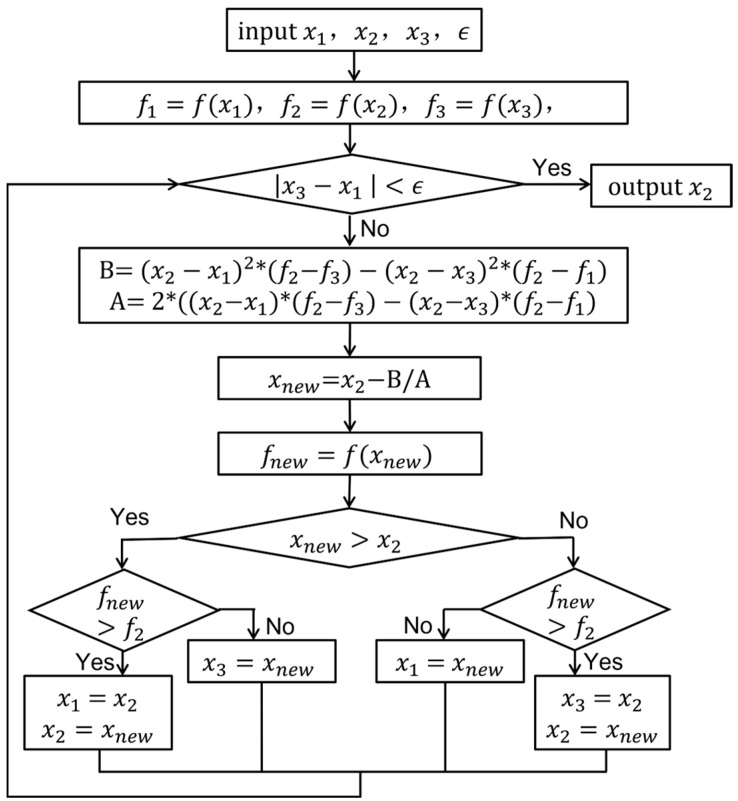
Flow chart of the parabolic interpolation method.

The flowchart for realizing the parabolic interpolation method is shown in Figure 5. The parabolic interpolation method converges faster, particularly near the peak point, and can quickly approximate the real peak. It utilizes the local characteristics of the function and can estimate the peak position more accurately for some functions with smooth characteristics. However, it is more sensitive to the selection of the initial point. If the initial point is not properly selected, the algorithm may converge to the local extreme value instead of the global maximum value. Each iteration must solve a system of ternary equations, and the calculation is relatively large.

#### 2.2.3. Brent Method

The Brent method combines the advantages of the golden section method and the parabolic interpolation method and is an efficient algorithm for searching for the extreme values of one-dimensional functions [33,34]. It automatically uses the golden section method or parabolic interpolation method according to the characteristics of the function during the iteration process. When the function shows better smoothness in the current interval, the parabolic interpolation method is used for fast approximation, and when the function changes are more complex and it is difficult to approximate them accurately with a parabola, the golden section method is used to ensure the stability of the algorithm.

In the fractional frequency domain peak search, the Brent method first determines an initial interval $\left[a,b\right]$ containing the peak points and then selects an initial trial point $x_{0}$ within the interval. During the iteration process, on the basis of the comparison of the function values and the current interval, it is decided whether to use the parabolic interpolation method to update the trial point or the golden section method to narrow the interval.

Conditions for the use of parabolic interpolation: When the function satisfies certain smoothness conditions in the current interval $\left[a,b\right]$, Brent method uses parabolic interpolation. The Brent method considers the following three guidelines when choosing whether to use the golden section method or parabolic interpolation method.

(1) Parabolic interpolation is used if the quadratic parabola constructed from the three existing points $x_{1}$, $x_{2}$, and $x_{3}$ in the current interval fits the original function well; that is, the fitting error is within an acceptable range. The effectiveness of the fit can usually be measured by determining the deviation of the function value of the parabola at these three points from the value of the original function, which is calculated as follows:

(10) $$\max(|f(x_{1})-y(x_{1})|,|f(x_{2})-y(x_{2})|,|f(x_{3})-y(x_{3})|)$$

If this value is less than a predetermined threshold δ, the function is considered smooth in the interval and suitable for parabolic interpolation. Additionally, the distribution of the three points must be relatively uniform to ensure that the parabola accurately reflects the local trend of the function.

(2) In the process of constructing the parabola, the coefficients are found to be abnormal (e.g., the denominator tends to 0), which indicates that the function is not suitable for parabolic approximation and then switches to the golden section method.

(3) When there is a trial point beyond a reasonable range in the iterative process or when the new peak estimation point of the parabolic method is not larger than the original estimation value, the golden section method is also used to ensure the stability of the algorithm and gradually reduce the interval to avoid dispersion of the algorithm owing to the parabolic interpolation method.

The Brent method synthesizes the advantages of the golden section method and parabolic interpolation method, which have faster convergence speeds and ensure the stability of the algorithm. It exhibits good adaptability to various types of functions and can accurately determine the peak point in different situations.

### 2.3. Research on the Anti-Frequency Sweeping Jamming Method That Is Based on the Fractional Domain Matching Order

LFM radar plays a key role by virtue of its unique advantages: its detection mechanism transmits signals with special waveform, receives the echoes reflected back from the target, and then processes the echoes to obtain relevant information about the target.

Equation (3) is the expression for the LFM radar transmission signal. Assuming that K targets exist in the radar monitoring area, the target echo signal can be expressed via the following equation:

(11) $$s_{re}(t)=\sum_{k=1}^{K}A_{k}exp\left\{j2\pi \left[f_{0}\left(t-\tau_{k}\right)+0.5\mu {\left(t-\tau_{k}\right)}^{2}\right]\right\},0<t<T$$

where $A_{k}$ indicates the target k corresponding to the echo signal amplitude, the size of which is affected by a variety of factors, such as the target’s radar scattering cross-sectional area, which reflects the target’s ability to scatter radar signals; the larger the scattering cross-sectional area is, the larger the echo amplitude; and the distance between the target and the radar, the further the distance, the greater the energy attenuation of the echo in the process of propagation, the smaller the amplitude, and the propagation process of the loss. $\tau_{k}$ is the time delay of the target k echo signal, which is closely related to the distance $R_{k}$ from target k to the radar and satisfies $\tau_{k}=2R_{k}/c$ (c for the electromagnetic wave propagation speed). This echo signal is actually a superposition of multiple target echoes, each of which carries its own information related to the radar distance, and this information becomes an important basis for the radar to detect the location of the target.

After receiving the target echo signal, the radar mixes it with the transmitted signal and is then filtered by a bandpass filter, finally outputting the target echo difference frequency signal carrying the target distance and speed information.

(12) $$\begin{array}{l} s_{re\_if}(t)=bpf\left[s_{tr}(t)s_{re}^{*}(t)\right]\\ =\sum_{k=1}^{K}A_{k}exp\left[j2\pi \left(f_{0}\tau_{k}+\mu \tau_{k}t+0.5\mu \tau_{k}^{2}\right)\right] \end{array}$$

Here, bpf(⋅) represents the bandpass filter operation, which allows only the signals of a specific frequency range to pass through, filtering out the interfering signals of other frequencies. $s_{re}^{*}(t)$ is the conjugate of the target echo signal $s_{\mathrm{re}}\left(t\right)$. By analyzing the expression of the difference frequency signal, it can be determined that it is the sum of several single-tone signals, and the frequency of each single-tone signal implies the distance and speed information of the target. In practical applications, the difference frequency signal is usually processed via Fourier transform (FT), and the distance information of the target can be deduced according to the position where the peak of the spectrum of the difference frequency signal appears and combined with the relationship $\tau_{k}=2R_{k}/c$. In an actual electromagnetic environment, radar may be subject to interference. The swept interference signal generated by the jammer can be modeled via the following equation:

(13) $$s_{in}(t)=A_{in}exp\left\{j2\pi \left[f_{in}t+0.5\mu_{in}t^{2}\right]\right\},0<t<T_{in}$$

where $s_{in}\left(t\right)$ represents the amplitude of the jamming signal, which directly reflects the intensity of the jamming signal; $f_{in}$ is the carrier frequency of the jamming signal, which determines the center frequency position of the jamming signal in the spectrum; $\mu_{in}$ is the modulation slope of the jamming signal, which controls the rate of change in the frequency of the jamming signal with respect to time; and $T_{\mathrm{in}}$ is the modulation period of the jamming signal. In addition, the carrier frequency $f_{in}=f_{in0}+i\Delta f_{slope}$ of the interference signal, where i is the frequency point serial number (i=1,2,⋯), $f_{in0}$ is the initial frequency, and $\Delta f_{slope}$ is the frequency step value, together determine the frequency-change rule of the interference signal. The purpose of the frequency-sweeping interference signal is to interfere with the normal detection of the target by the radar, destroying the characteristics of the echo signal received by the radar and making it difficult for the radar to identify the target accurately. Therefore, the study of anti-frequency sweeping interference algorithms is particularly critical.

When an interfering signal enters the radar system, the interfering difference frequency signal obtained after mixing and bandpass filtering can be expressed as:

(14) $$\begin{array}{l} s_{in\_if}(t)=bpf\left[s_{in}(t)s_{re}^{*}(t)\right]\\ =bpf\left\{A_{in}exp\left[j2\pi \left(f_{0}-f_{in}\right)t+j\pi \left(\mu -\mu_{in}\right)t^{2}\right]\right\},0<t<T \end{array}$$

Assuming that the instantaneous frequency of the interference signal enters the fuzing bandpass filter at moment $\tau_{1}$ and leaves the bandpass filter at moment $\tau_{2}$, the interference difference frequency signal can be further simplified as

(15) $$\begin{array}{l} s_{inj}(t)=rect\left(t-\tau_{1},\tau_{1}-\tau_{2}\right)exp\left\{j2\pi \left[f_{lps}\left(t-\tau_{1}\right)+0.5\left(\mu -\mu_{in}\right){\left(t-\tau_{1}\right)}^{2}\right]\right\},\\ 0<t<T \end{array}$$

where $rect(\tau_{1}-\tau_{2})$ is the gate function with a pulse width of $\tau_{1}-\tau_{2}$, which limits the effective range of action of the interference signal in time, and where $f_{\mathrm{lps}}$ is the starting frequency of the passband of the bandpass filter. The interference difference frequency signal is an initial frequency $f_{0}-f_{\mathrm{in}}$, the modulation slope $\mu -\mu_{in}$ of the LFM signal, and its special frequency characteristics interfere with the normal sensing of the radar, making it difficult for the radar to accurately distinguish the target signal from the echo.

In the case of frequency sweeping interference, the final output of the fuzes $S_{if}\left(t\right)$ is the superposition of the target echo difference frequency signal, interference difference frequency signal, and environmental noise.

(16) $$\begin{array}{l} s_{if}(t)=\sum_{k=1}^{K}A_{k}exp[j2\pi \left(f_{0}\tau_{k}+\mu \tau_{k}t+0.5\mu \tau_{k}^{2}\right]\}\\ +rect\left(t-\tau_{1},\tau_{1}-\tau_{2}\right)exp\left\{j2\pi \left[f_{lps}\left(t-\tau_{1}\right)+0.5\left(\mu -\mu_{in}\right){\left(t-\tau_{1}\right)}^{2}\right]\right\}\\ +N(t),0<t<T \end{array}$$

In actual radar-sensing scenarios, the difference in the frequency signal under swept interference becomes very complex. The energy of the interfering signal may be much greater than that of the target echo difference frequency signal, resulting in the target echo signal being easily overwhelmed by the interfering signal. For example, in a strong interference environment, the spectral components of the difference frequency signal received by the radar are dominated by the spectral components of the interference signal, and the spectral characteristics of the target echo signal are difficult to recognize, making accurate detection of the target’s distance and speed information via conventional signal processing methods difficult. Therefore, to ensure that the radar can operate normally in an interference environment, it is necessary to study and adopt the corresponding anti-interference technology [35,36,37] to suppress the influence of the interference signal and accurately extract the target information.

According to the definition of the FRFT, the FRFT of the LFM signal after time delay τ is

(17) $$\begin{array}{l} F_{p}[x(t-\tau )]=\sqrt{\frac{1-j\cot\alpha }{2\pi }}e^{\frac{ju^{2}\cot\alpha }{2}}\cdot \int_{-T}^{T}x(t-\tau )e^{j(\frac{1}{2}t^{2}\cot\alpha -ut\csc\alpha )}dt\\ =A\sqrt{\frac{1-j\cot\alpha }{2\pi }}e^{\frac{ju^{2}\cot\alpha }{2}}e^{-j(f_{0}\tau +\frac{1}{2}\mu \tau^{2})}\cdot \int_{-T}^{T}e^{j\frac{\left(\cot\alpha -\mu \right)}{2}t^{2}+j(f_{0}+\mu \tau -u\csc\alpha )t}dt \end{array}$$

When $\alpha_{0}=\arctan(-1/\mu )$, substituting into the above equation yields:

(18) $$\left|F_{p_{0}}[x(t-\tau )]\right|=2AT\sqrt{\frac{\csc\alpha_{0}}{2\pi }}\left|\sin c[(f_{0}+\mu \tau -u\csc\alpha_{0})T]\right|$$

The function sinc includes the time delay τ in the independent variables, which means that the time delay affects the shape of the amplitude spectrum. When $f_{0}+\mu \tau =u\csc\alpha_{0}$, the maximum value of $\left|F_{p_{0}}[x(t-\tau )]\right|$ is reached. As the equation contains the time delay τ, the location of the peak point is related to the delay time τ. If the LFM signal is delayed by $\tau_{1}$ and $\tau_{2}$, the difference in the location of the peak point of its FRFT mode function is

(19) $$u_{2}-u_{1}=\mu (\tau_{2}-\tau_{1})\sin\alpha_{0}=(\tau_{2}-\tau_{1})\cos\alpha_{0}$$

In the expression of $\left|F_{p_{0}}[x(t-\tau )]\right|$, $2AT\sqrt{\frac{\csc\alpha_{0}}{2\pi }}$ does not include the time delay τ; therefore, the peak size of the FRFT amplitude spectrum of the LFM signal after the time delay is independent of the time delay.

In the best FRFT domain of the difference frequency signal, the interference signal is gathered as a spike, and the fuzed echo signal is a single-frequency signal. Since the transform order is not optimal for the fuzed signal, the FRFT domain spectrum does not exhibit spiky characteristics. Thus, the band-stop filter can be used to filter out the spike where the interference signal is located to realize anti-jamming and correctly extract the target position information.

Furthermore, according to the optimal transform order of the difference frequency signal to determine the interference signal chirp rate, a copy of the peak position-matched interference signal is constructed, the copy and the return difference frequency signal cancellation are used, and residual spike filtering is performed. The FRFT domain signal can be obtained after de-interfering the signal. The signal for fractional-order Fourier inversion can be obtained, and the return difference frequency signal in the time domain can be obtained after de-interfering the signal. The target position information can be obtained by using the spectral peak position of the signal.

## 3. Simulation and Performance Comparison

### 3.1. Simulation and Performance Comparison of Three Search Algorithms

The golden section method only needs to calculate the function values of two points per iteration, with lower computational complexity; the parabolic interpolation method needs to solve a system of ternary equations per iteration, with a larger amount of computation; and the Brent method, owing to the need to choose a different method according to the function situation, has computational complexity between the two, and may require more computational resources in some cases.

The golden section method has certain requirements for the selection of the initial interval, but as long as the initial interval contains the peak point, it can converge normally. The parabolic interpolation method is more sensitive to the selection of the initial point, and improper selection of the initial point may lead to convergence to local extremes. The Brent method is relatively less sensitive to the initial conditions, and it can converge to the global maximum better under different initial conditions.

The simulation chirp rate was 250 kHz/s for the LFM signal, and its theoretical best FRFT order was 1.157. As shown in Table 1, the Brent method had the highest accuracy, and the relative error was always small and stable. The parabolic interpolation method has good accuracy when the convergence threshold is small, such as when the relative error is small when the convergence threshold is 10^−3^, but the error increases significantly when the convergence threshold becomes large and when the stability is poor. The relative error of the golden section method was greater than that of the other methods, and its accuracy was the lowest among the three methods. The Brent method integrates multiple strategies and can more accurately approach the optimal solution. The parabolic interpolation method relies on quadratic fitting and is sensitive to changes in the convergence threshold. The golden section method has a relatively single search strategy, and it is difficult to accurately lock the optimal solution.

The parabolic interpolation and Brent methods are more efficient, and the number of iterations is relatively small. The Brent method has only six iterations when the convergence threshold is 10^−2^, which is an obvious advantage. The golden section method has several iterations, long calculation times, and low efficiency. The parabolic interpolation method uses the local quadratic characteristics of the function to accelerate the search, and the Brent method integrates multiple efficient strategies to dynamically adjust the search method, whereas the golden section method narrows the interval according to a fixed ratio, and the search process is slow.

**Figure 6:**
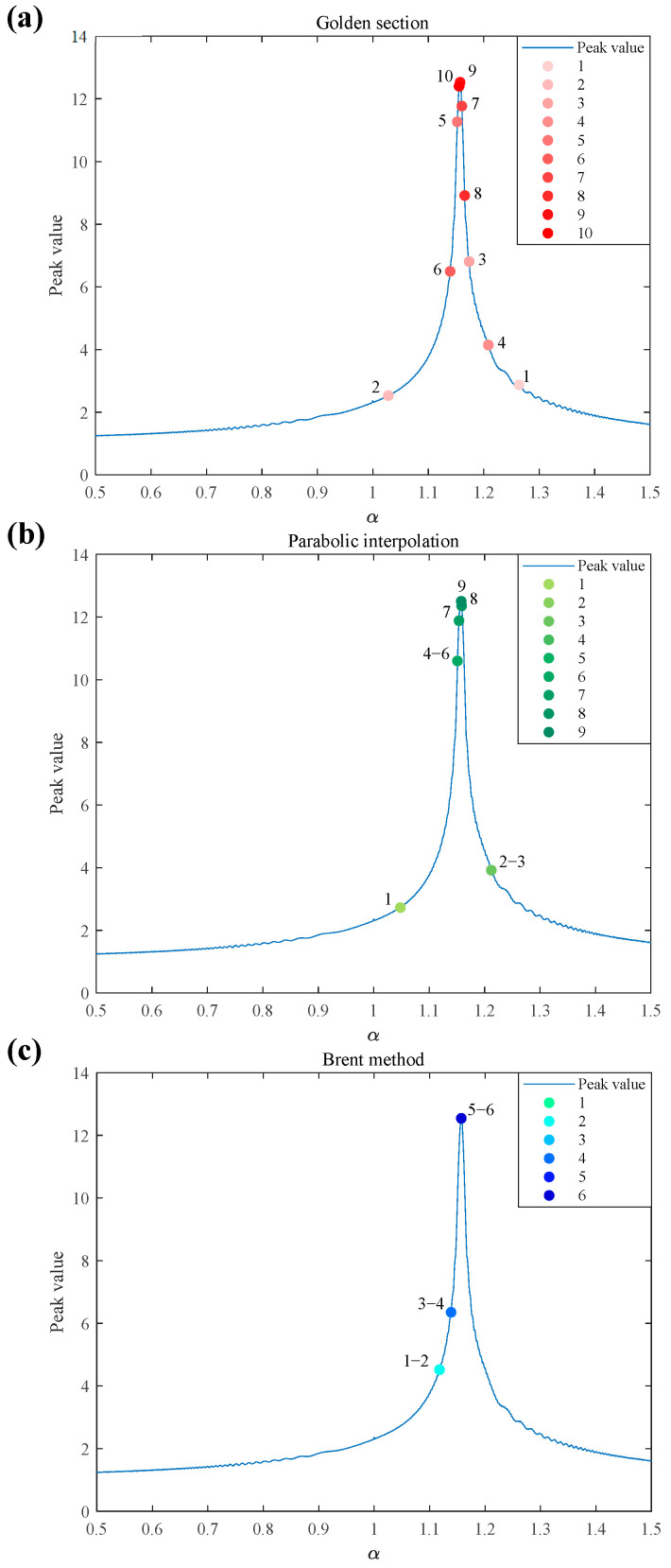
The course of the three peak search algorithms. (**a**) Golden section; (**b**) parabolic interpolation; (**c**) Brent method.

As shown in Figure 6, the golden section method was close to the peak position in 10 iterations. The distribution of the red dots in the iteration process is more scattered, which indicates that in the search process, the narrowing of the search interval in each iteration is relatively slow, and the convergence speed is slower. The parabolic interpolation method approached the peak position in the 9th iteration. Compared with the golden section method, it can approach the peak value faster and locate a better position with fewer iterations, indicating that its convergence speed is relatively fast. The Brent method approaches the peak position in the 5th to 6th iterations. Clearly, it finds the peak neighborhood faster than the first two methods do, and its convergence speed is the fastest among the three methods.

The relatively uniform distribution of red dots in the iteration process of the golden section method indicates that it explores the function relatively smoothly during the search process, is less affected by the local characteristics of the function, and has better stability but at the cost of slow convergence. The figure shows that the distribution of red dots in the parabolic interpolation method also has a certain regularity, and the overall stability is still good. However, in some iteration processes, owing to the quadratic fitting characteristics of the function, it is affected by the local curvature of the function changes, etc., and the stability is slightly inferior to that of the golden section method. The Brent method combines a variety of search strategies, and it converges to the vicinity of the peak value well under the characteristics of some complex functions.

### 3.2. Integrated Frame Design

**Figure 7:**
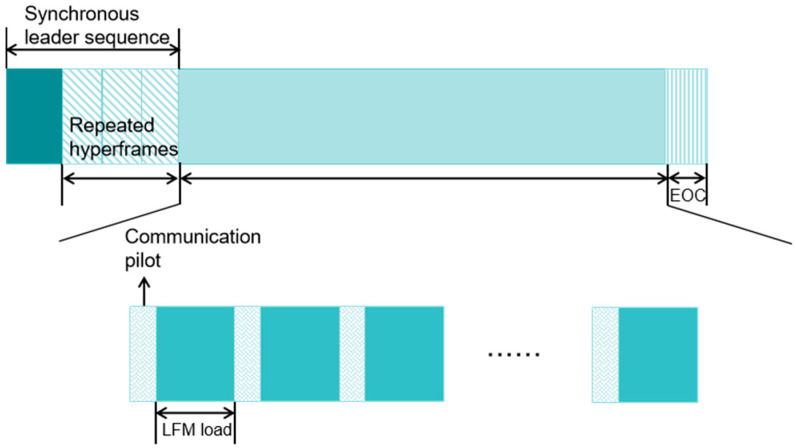
Integrated frame structure.

The integrated frame design is shown in Figure 7. This design adopts the LFM signal with a fixed chirp rate as the synchronization head, which has good autocorrelation characteristics, and its linear frequency change in the time domain enables the receiver to capture the starting position of the signal quickly and accurately through correlation operation to realize symbol synchronization. Meanwhile, by analyzing the frequency variation in the LFM signal, the frequency offset of the signal can be estimated initially to complete the coarse frequency offset estimation.

The communication pilot helps the system estimate the transmission characteristics of the channel. Due to the complexity of the wireless channel, the signal will be affected by the multipath effect, and it will fade during transmission, etc. The pilot signal can be used as a reference for the receiver to understand the change in the channel so that the received signal can be corrected and compensated to improve the accuracy of communication.

The LFM data segment transmits information through chirp rate coding, and each symbol is able to map four chirp rate values, corresponding to a bandwidth of 3, 6, 9 and 12 kHz, respectively. Selecting the chirp rate values and bandwidths, as well as rationally allocating the guide frequency and the length of the data segment, ensures that the information can be transmitted accurately and reliably. Since the information is transmitted through the frequency change in the signal instead of simple amplitude or phase modulation, it is better able to resist noise and interference and ensure the accurate transmission of information in complex electromagnetic environments.

### 3.3. LFM Load Performances Simulation

Parabolic interpolation is based on quadratic polynomial fitting, which approximates a complex original function with a relatively simple quadratic curve. When there are multiple extreme points in the original function, the fitted quadratic parabola may not accurately reflect the true shape of the original function over the entire search interval. In addition, the performance of the parabolic interpolation method was highly sensitive to the initial three points selected. If the initial three points are located in a local region of the function and the function shape in this region is more complicated (e.g., there are multiple extreme points or the function changes drastically), then the fitted parabola may be affected by the limitations of these points, which causes the extreme point of the parabola to fall at a local extreme value. In addition, if the initial point is far from the global extreme value point during the iteration process because the parabola is fitted on the basis of the three local points each time, the algorithm may loop in the local region and cannot jump out and find the global optimal solution, thus falling into the local extreme value.

Parabolic interpolation is a local search method that considers only the information of the three points localized within the search interval to construct an approximation model. This local approximation lacks the knowledge and understanding of the global properties of the function. During the iteration process, the algorithm only updates the search points on the basis of the current local extreme parabolic points without fully considering the trend of the function over a wider range. When the trend of the function in the local region misleads the fitting direction of the parabola, the algorithm can easily converge to the local extreme value and miss the global optimal solution.

**Figure 8:**
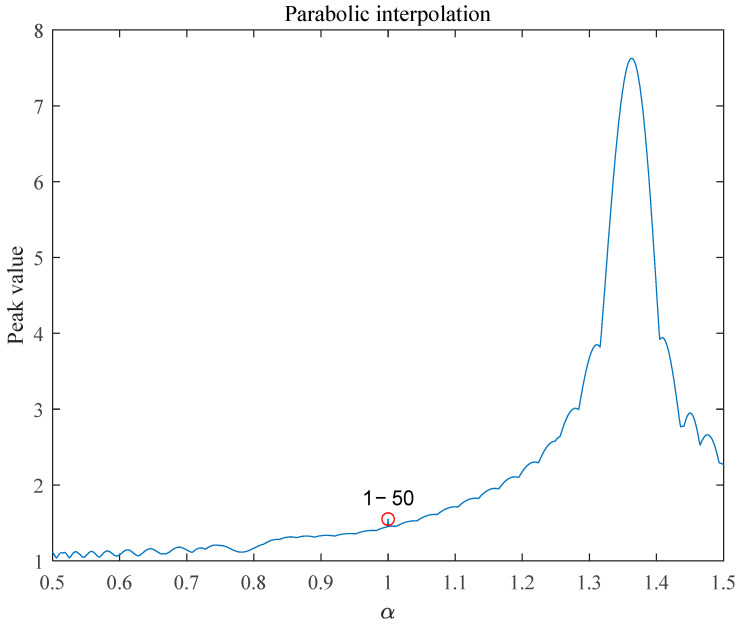
Parabolic interpolation plunging into local maximum for bandwidth of 12 kHz.

Table 2 shows the estimated order and the number of iterations of the three search algorithms for different bandwidths in the integrated waveforms. When the signal bandwidths are 9k and 12 kHz, the number of iterations of the parabolic interpolation reaches the set upper limit of 50, and the parabolic interpolation falls into the point of local maximum, as shown in Figure 8.

Compared with parabolic interpolation and the Brent method, the golden section search method approaches the extreme value point by continuously narrowing the interval, which is adaptable to the multipolar characteristics of the function; the Brent method combines the advantages of multiple methods, which can address different situations more flexibly during the search process and is less likely to fall into the local maxima than the parabolic interpolation method is. Therefore, in the LFM load communication bit error ratio (BER) simulation, we used only the golden section search method and the Brent method.

**Figure 9:**
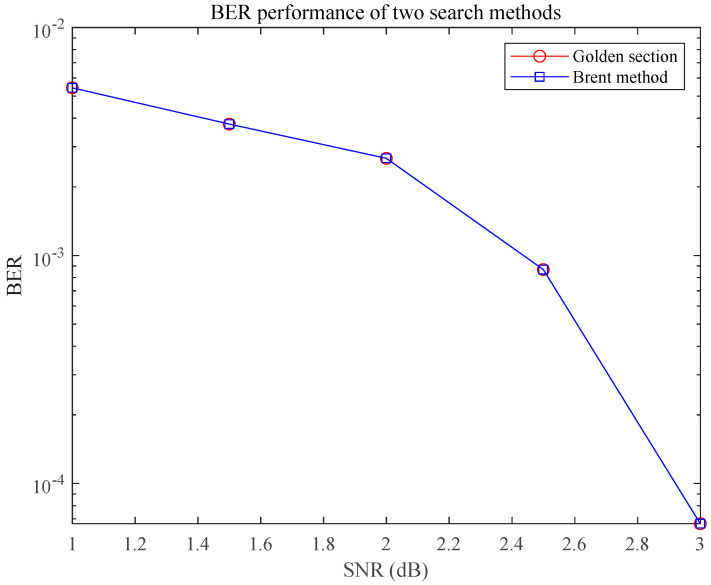
BER performance of the golden section method and Brent method.

To verify the communication performance of the signal, we simulate the communication performance of the LFM load, using only the golden section search method and the Brent method in the demodulation of the LFM frequency. The Monte Carlo simulation was performed 1000 times to determine the average value of the BER and compare it, and the results are shown in Figure 9.

The figure shows that the BERs of the golden section search method and Brent method for chirp rate demodulation also decrease with increasing SNR, and the BERs of the two loads meet the requirements of the communication system and are capable of meeting the working conditions of shortwave communication. The Brent algorithm proposed in this study has fewer iterations than the golden section method in the case of guaranteeing communication performance and has higher agility.

**Figure 10:**
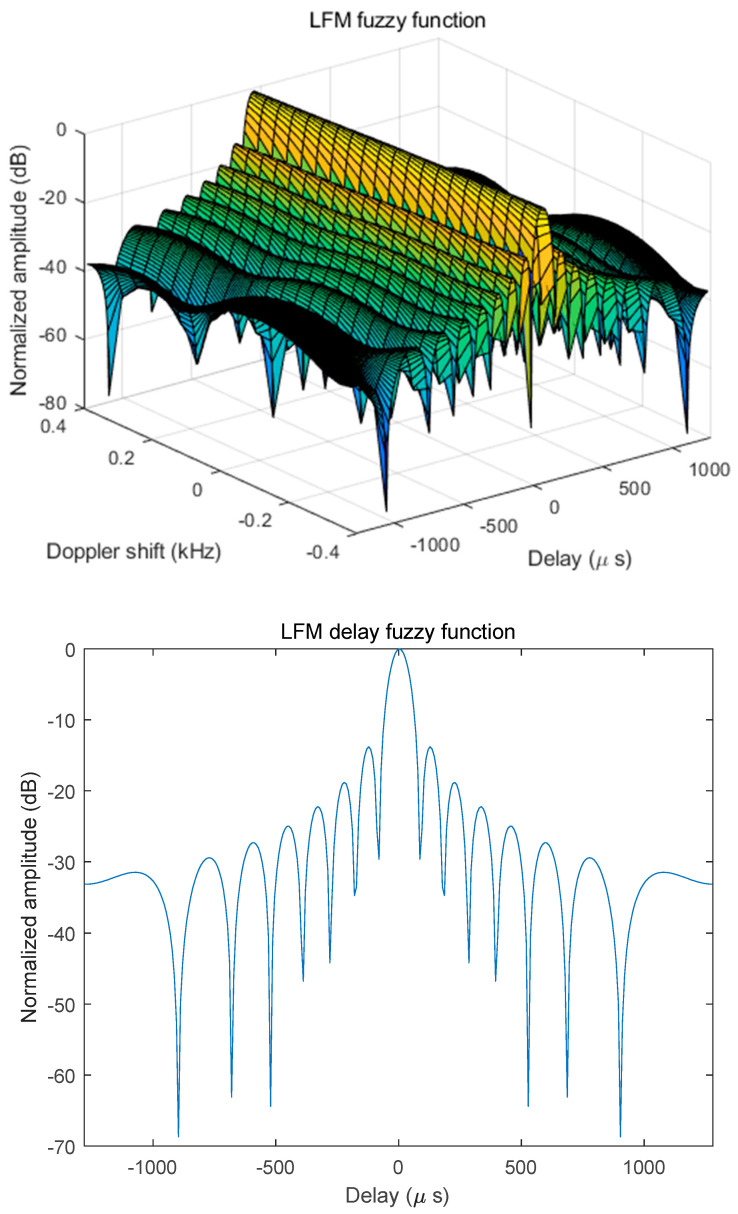
The fuzzy function and time delay for this LFM signal at 12 kHz bandwidth.

The fuzzy function and time-delay fuzzy of this LFM signal under the simulated 12 kHz bandwidth are shown in Figure 10, in which the LFM time-delay fuzzy function has an obvious main peak at time-delay 0, which indicates that the target time-delay can be accurately estimated in the ideal state, and then the target distance can be accurately calculated. The relatively low sidelobe ensures the resolution of the distance estimation and meets the detection requirements for target localization accuracy. In summary, this integrated design can realize the communication function by ensuring distance detection performance.

### 3.4. Anti-Frequency Sweeping Interference Performance Simulation

**Figure 11:**
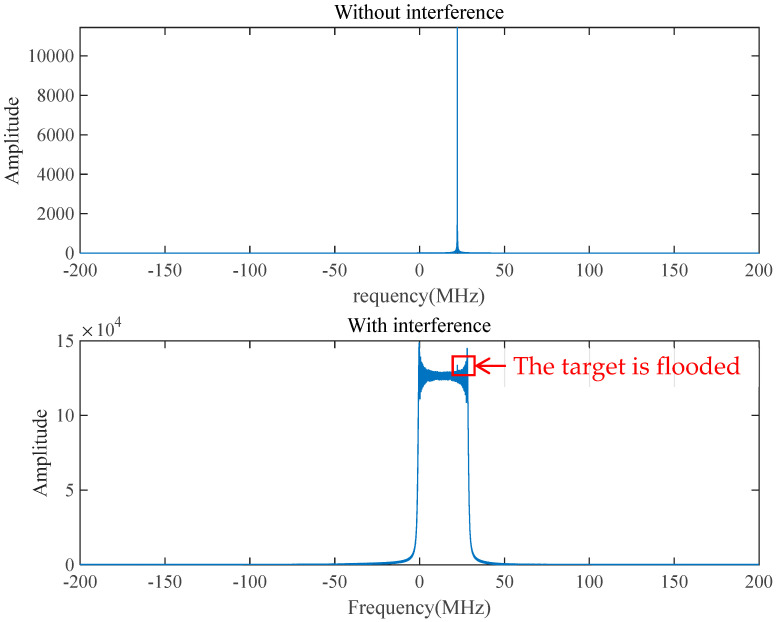
Spectrum of the difference frequency signal under sweeping interference.

By simulating the spectrum of the difference frequency signal under swept interference with the above parameters below in Table 3, it can be seen in Figure 11 that the frequency of the echo signal carrying the target distance information is submerged in the band of the interfering signal, and the target is not recognized.

**Figure 12:**
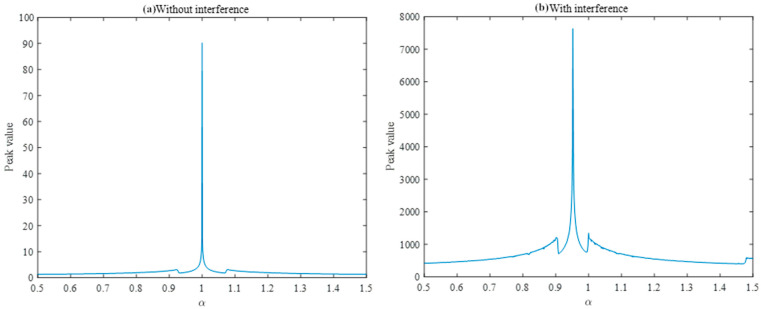
FRFT peaks of two different frequency signals.

Fractional-order Fourier analysis is performed on the interference-free difference frequency signal and interference difference frequency signal, and the best FRFT order is found by using the aforementioned golden section search method and the Brent method. The optimal order FRFT is adopted for the two signals respectively as shown in Figure 12. The best FRFT order of the interference-free difference frequency signal is 1, which is the traditional Fourier transform according to the definition of the FRFT, and the frequency domain information can be obtained. According to Equation (12), the interference-free difference frequency signal is a single-frequency signal, and its optimal FRFT is the conventional Fourier transform. According to Equation (16), the interference difference frequency signal is a single-frequency signal superimposed on an LFM signal, and the chirp rate is the difference between the fuse chirp rate and the interference chirp rate. Thus, the difference between the fused chirp rate and the interference chirp rate can be determined by order of the best FRFT of the interference difference frequency signal, and the difference between the fuse chirp rate and the interference chirp rate can be obtained because the fuse chirp rate is known to the system.

**Figure 13:**
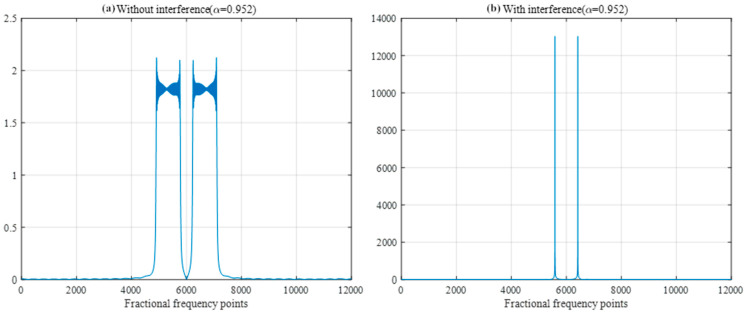
Optimal FRFT results with interfering difference frequency signals.

Use the golden section search method and Brent method to find the optimal fractional order of the transform for the interfering difference-frequency signal. Under the simulation parameters used in Table 3, the best FRFT order obtained by the two search methods is 0.9523, as shown in Figure 13. The difference frequency signal presents two spikes in the best FRFT order domain; the location of the spikes is related to the chirp rate of the interference signal, and the amplitude of the spikes is related to the energy of the interference signal. By simultaneously simulating the ideal difference frequency signal without interference under the same order transform, its FRFT domain spectrum does not show spike characteristics, and the peak amplitude is very low compared with the interfering difference frequency signal.

**Figure 14:**
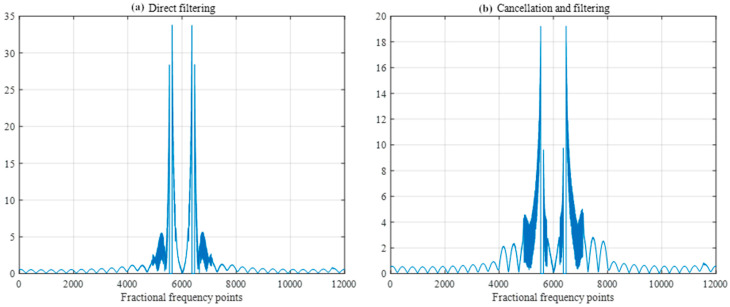
FRFT spectrum after direct and pairwise filtering.

**Figure 15:**
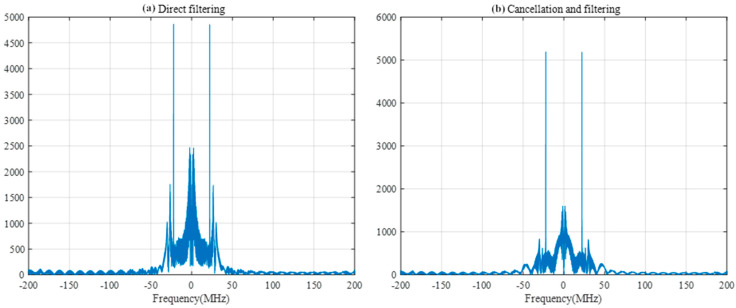
Frequency spectrum after direct filtering and pairwise filtering.

The width of the filters was set to 50 sample points, and the SIR was −40 db. A comparison of the FRFT spectrum as shown in Figure 14 and the frequency spectrum as shown in Figure 15 after direct filtering and pairwise cancellation filtering reveals that the peak of the FRFT spectrum after pairwise cancellation filtering has a lower peak, which is closer to the characteristics of the ideal difference frequency signal without interference. The peak positions of the frequency spectrum obtained by the two methods are consistent with the peak positions of the frequency spectrum of the interference-free difference frequency signal in Figure 11, and both methods can correctly detect the target distance.

In radar signal processing, the peak–sidelobe ratio (PSLR) is used to measure the relative magnitude of the relationship between the peak of the main flap and the peak of the largest sidelobe in the signal spectrum via the expression

(20) $$PSLR=10\log_{10}\left(\frac{P_{main}}{P_{sidelobe}}\right)$$

where $P_{main}$ denotes the peak power of the main flap in the spectrum, that is, the peak power reflecting the target distance information, and where $P_{sidelobe}$ denotes the maximum peak power of the side flap in the spectrum in addition to the main flap.

**Figure 16:**
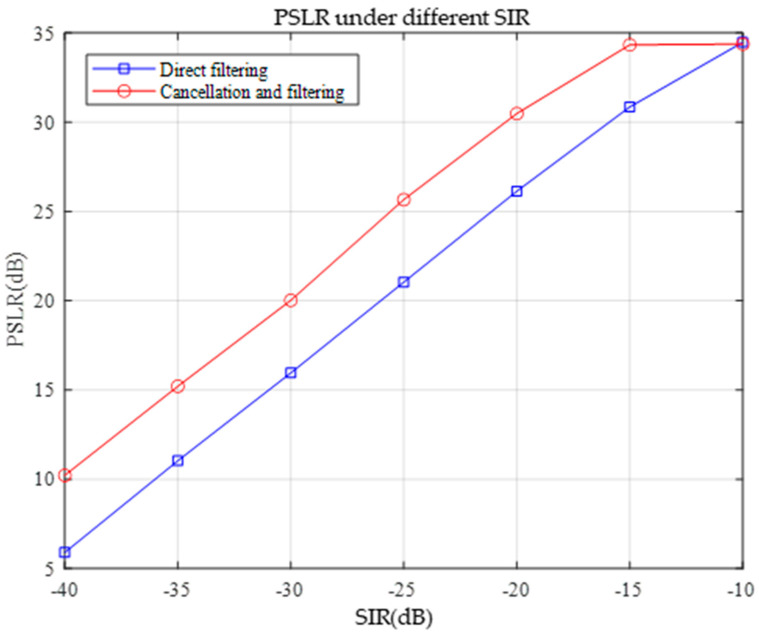
Frequency-domain PSLR after direct and pairwise filtering.

In the context of anti-sweeping interference, a good anti-interference algorithm should make the target signal more prominent in the spectrum while the interference signal is effectively suppressed. A higher PSLR value means that the peak power of the main flap is greater than the maximum peak power of the side flap, i.e., the power difference between the main flap and the side flap is more significant. At this time, the peak value of the target signal is more obvious in the spectrum, and in the detection process, it is easier to distinguish the target signal from the background noise and the interference signal, thus reducing the leakage detection rate. Thus, PSLR can effectively evaluate the performance of the anti-interference algorithm. The performance of the two methods was evaluated under different signal-to-noise ratios. The simulation results in Figure 16 indicate that the PSLR values obtained via the pairwise cancellation filtering method are always higher than those obtained via the direct filtering method under different SIR conditions. This finding indicates that pairwise cancellation filtering performs better in suppressing the sidelobes and can make the peaks of the target signal more prominent in the spectrum with respect to the sidelobes. According to the principle of PSLR to evaluate the anti-interference performance, a higher PSLR value means that the pairwise cancellation filtering method can reduce the leakage detection rate more effectively, the anti-interference ability is stronger in the case of a low SIR, and it has a better performance in the anti-interference of frequency sweeping. Therefore, summarizing the simulation results, the pairwise cancellation filtering method is better than the direct filtering method for the application of anti-sweep interference based on LFM radar.

**Figure 17:**
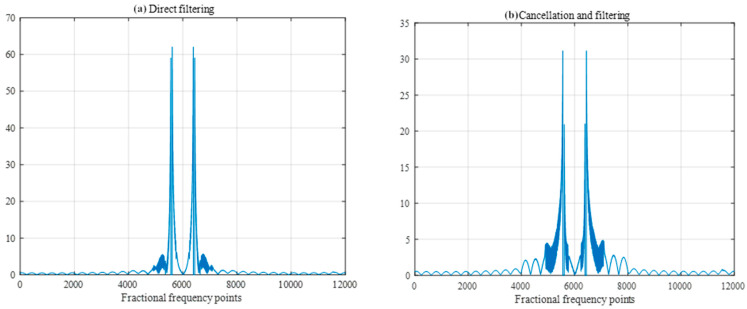
FRFT spectrum after direct and pairwise filtering with a filter width of 25 samples.

**Figure 18:**
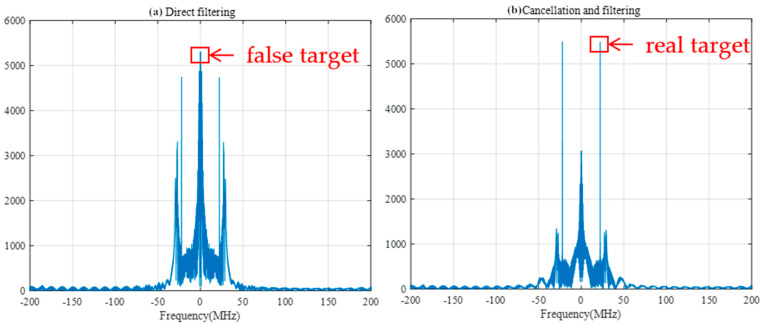
Frequency spectrum after direct and pairwise filtering with a filter width of 25 samples.

Next, the performances of direct filtering and pairwise cancellation filtering under different filter widths are discussed. The filter width is set to 25 samples, and the SIR is kept at −40 db. When the FRFT spectrum as shown in Figure 17 and frequency spectrum as shown in Figure 18 after direct filtering and pairwise cancellation filtering are compared, the peaks of pairwise cancellation filtering can correctly reflect the location of the target since the pairwise cancellation filtering method can first carry out pairwise cancellation processing on the interference signal, which purposefully attenuates the effect of frequency sweeping interference on the target signal. The maximum peak of direct filtering is near the 0-frequency point, in which case the energy of the interfering signal may mask the true peak of the target signal, causing the maximum peak to appear at the wrong location, thus affecting the accurate judgment of the target location.

**Figure 19:**
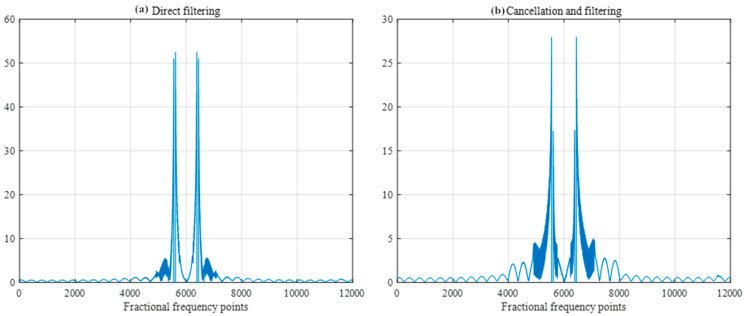
FRFT spectrum after direct filtering and cancellation filtering with a filter width of 30 samples.

**Figure 20:**
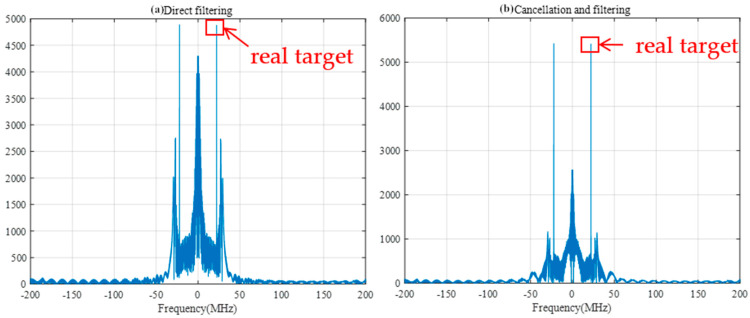
Frequency spectrum after direct and pairwise filtering with a filter width of 30 sample points.

When the SIR is −40 dB, the width of the filter is set to 30 sample points, and the FRFT spectrum as shown in Figure 19 and frequency spectrum as shown in Figure 20, after direct filtering and pairwise cancellation filtering, are compared. At this time, the peak position of the spectrum obtained by the two methods is the same as the peak position of the spectrum of the difference frequency signal under no interference in Figure 11, and both methods can correctly detect the distance to the target. However, the amplitude near the 0-frequency point in the direct filtering method is relatively high and may be mistakenly detected as a target.

**Figure 21:**
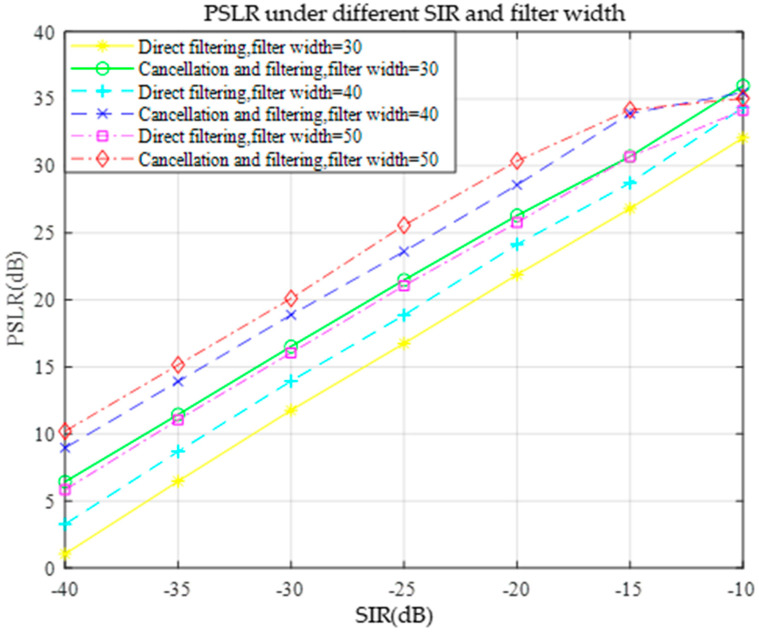
PSLR with different SIR and filter widths.

Under an SIR of −40 dB, when the filter width is greater than 30 sample points, both direct filtering and pairwise cancellation filtering can theoretically detect the target distance correctly, and the PSLR is extended as the evaluation index of the anti-jamming performance to evaluate the composite effect of the simulated SIR and filter width on the anti-jamming performance. Under the conditions of different SIR and filter widths, the PSLR of the two methods of direct filtering and pairwise cancellation filtering was simulated as shown in Figure 21, and the PSLR was used as an index for evaluating the anti-jamming performance; the higher the value, the better the anti-jamming performance.

The figure shows that the PSLR value tends to increase with increasing SIR, whether it is direct filtering or pairwise cancellation filtering. This is because a higher SIR means that there are relatively fewer interference components in the signal and that the target signal is more prominent. In this case, the filtering operation enhanced the target signal more effectively and suppressed the side flaps, resulting in an increase in the PSLR value. When the filter width is fixed, the PSLR values of both filtering methods increase significantly as the SIR gradually increases from −40 dB to −10 dB, indicating that the interference immunity increases with increasing SIR.

For direct filtering, as the filter width increases from 30 to 50 sample points, the PSLR value gradually increases at the same SIR. This is because a wider filter can suppress the interference components more adequately, thus enhancing the power ratio between the main and side flaps of the target signal. However, for pairwise cancellation filtering, although the PSLR value increases with increasing filter width, the increase is smaller than that of direct filtering. This may be because pairwise cancellation filtering itself significantly improves the anti-interference performance through the operation of canceling the interfering signals, and the marginal effect of the increase in filter width on its performance improvement is relatively small.

For the same SIR and filter width, the PSLR value of pairwise cancellation filtering was always greater than that of direct filtering. This finding indicates that pairwise cancellation filtering performs better at suppressing interference and improving the clarity of the target signal. Even at a lower SIR of −40 dB, the pairwise cancellation filter still achieves a relatively high PSLR value, indicating that it is more capable of suppressing interference. In addition, when the filter width is greater than 30 samples, and the SIR is sufficiently high, both methods can theoretically detect the target distance correctly; however, the PSLR value of the pairwise cancellation filter is higher, which means that its anti-interference performance is superior, and the reliability of the detection results is also greater.

## 4. Discussion

This study aims to solve the problems of insufficient agility of parameter estimation methods and target detection under frequency-sweeping interference in integrated sensing and communication systems. The fast estimation of LFM signal parameters is realized by introducing the FRFT combined with the golden section, parabolic interpolation, and Brent methods. The designed LFM signal was successfully verified for the cooperative realization of the communication and sensing functions. The core findings include the following: the Brent method improves the parameter searching efficiency by approximately 30% compared with the golden section method; the BER of the integrated LFM signal can reach 10^−4^ with an SNR of 3 dB; and the pairwise cancellation filtering suppresses the frequency sweeping interference by 4–6 dB more than the direct filtering with an SIR ≤ −15 dB. Both filtering methods can maintain a PSLR of more than 10 dB when the SIR is as low as −30 dB.

Compared with the demodulation scheme based on the maximum likelihood (ML) detector proposed by Ahn et al. [38], this study reduced the communication BER of the LFM signal with an SNR of 2.5 dB to 0.087% (the traditional method is 2.2%) through the adaptive search strategy in the fractional-order domain. Compared with the FMCW radar anti-jamming scheme reported by Wang et al. [39], the cancellation and filtering method proposed in this study improves the PSLR by approximately 14 dB under the same −10 dB SIR.

The improvement in parameter estimation performance is due to the energy aggregation characteristic of the FRFT on the LFM signal. Combined with the global search ability of the golden section method, the Brent method effectively solves the problem of parabolic interpolation, easily falling into a local optimum. The advantage of pairwise cancellation filtering lies in its dual parallel structure: interference features are extracted by the reference branch, and the main branch realizes the cancellation superposition of interference signals. Further analysis shows that the time-varying characteristics of sweep interference affect the effect of direct filtering, whereas pairwise cancellation filtering maintains robustness to time-varying interference by dynamically updating the reference signal.

There are three limitations in this study:

(1) The performance of the parameter estimation method in a multipath channel environment has not yet been verified, and complex channels may lead to energy diffusion in the FRFT domain.

(2) The anti-interference experiment is only for linear sweep interference and does not involve complex interference types, such as comb interference.

(3) The communication performance under interference has not been analyzed yet. For integrated sensing and communication systems, interference can affect both communication and sensing functions.

On the basis of these limitations, the following work can be conducted in the future.

(1) Multiple path compensation algorithms, such as fractional-order domain equalization technology, are introduced to improve the accuracy of parameter estimation under complex channels.

(2) An adaptive interference recognition module is developed to realize intelligent classification and suppression of multiple types of interference.

(3) Discuss the impact of anti-jamming algorithms on communication performance and evaluate the impact of using filtering algorithms on the BER of LFM communication under different interference conditions. Hardware tests were performed to verify the algorithm’s real-time processing capability in an actual communication system. These studies will promote FRFT sensing and communication integration technologies for engineering applications.

## 5. Conclusions

With the widespread application of wireless communication and radar detection technologies, spectrum resources are becoming increasingly stringent, and the requirements for system integration and performance are increasing. As a key method to solve these problems, sensing and communication integration technology has become a research hotspot in academia and industry. In this study, the integration of sensing and communication was realized via LFM. The introduction of FRFT is an important part of this study. On the basis of the principle of the FRFT to realize LFM signal parameter estimation, the golden section method, parabolic interpolation method, and Brent method were applied to search for the best fractional-order domain. Moreover, an anti-interference method based on the fractional domain matching order is investigated. It utilizes the focusing of the interference component of the difference frequency signal in the FRFT domain, combined with the optimal fractional-order domain search algorithm, and adopts both direct filtering and pairwise cancellation filtering to achieve effective filtering of the interfering signals to detect the target distance, which enhances the performance of the system in a complex electromagnetic environment.

After simulation verification, the Brent method proposed in this paper is found to be better than the golden section method and parabolic interpolation method in terms of peak search agility and robustness. LFM carries communication information through chirp rate modulation coding, which has good BER performance. The two designed anti-sweeping interference methods can correctly extract the target location information under sweeping interference with a lower SIR and evaluate the detection performance of the two methods with different SIR and filter widths; the simulation results show that the performance of pairwise cancellation filtering is better than that of direct filtering. In conclusion, the integrated scheme of sensing and communication in this study is feasible and effective in both theory and practice.

Although this study demonstrates an integrated sensing and communication scheme, it is still necessary to optimize the system in combination with practical applications and obtain data feedback in real environments through field tests and experiments. On the basis of the problems and needs of practical applications, the system design was further optimized to improve the adaptability and stability of the system in complex electromagnetic environments, variable meteorological conditions, and different geographic environments. Moreover, it seeks multi-technology integration and synergistic development and actively explores integration with 5G and 6G technologies and new radar systems with the continuous development of communication and radar technologies. The high-speed and low-latency characteristics of 5G and 6G technologies provide new opportunities for the integration of sensing and communication.

## Figures and Tables

**Table 1:** Estimates and performances of the three search algorithms.

Search Method	Convergence Threshold	Number of Iterations	Computation Time (ms)	Estimated Order	Relative Error δp (%)
Golden Section	10^−3^	15	33.25	1.156681	0.0275
	10^−2^	10	61.67	1.156541	0.0397
Parabolic Interpolation	10^−3^	11	13.07	1.157055	0.0048
	10^−2^	9	13.24	1.157817	0.0706
Brent Method	10^−3^	11	23.44	1.157092	0.0080
	10^−2^	6	42.89	1.157098	0.0085

**Table 2:** Simulation of three search methods in an integrated waveform.

Search Method	Bandwidth	Number of Iterations	Estimated Order
Golden Section	3k	10	1.100813
	6k	10	1.196008
	9k	10	1.281153
	12k	10	1.358167
Parabolic Interpolation	3k	7	1.101283
	6k	8	1.197799
	9k	50	1.285511
	12k	50	1.000000
Brent Method	3k	6	1.100711
	6k	7	1.195048
	9k	6	1.281920
	12k	6	1.366473

**Table 3:** Simulation parameters of LFM radar sensing under frequency sweeping interference.

Simulation Parameters	Parameter Value
Fuse modulation period	30 µs
Fuse bandwidth	100 MHz
Target distance	1000 m
Interference bandwidth	130 MHz
Signal-to-interference ratio (SIR)	−40 dB

## Data Availability

Data will be made available on request.
